# Supplementary material for: Human cytomegalovirus regulates host DNA repair machinery for viral genome integrity
Source: Nucleic Acids Res. 2026 Jan 14;54(2):gkaf1445. doi: 10.1093/nar/gkaf1445 (PMC12802892; doi:10.1093/nar/gkaf1445)
Supplement: gkaf1445_Supplemental_File [file gkaf1445_supplemental_file.pdf]

Human cytomegalovirus regulates host DNA repair machinery for viral genome integrity

Supplementary Data

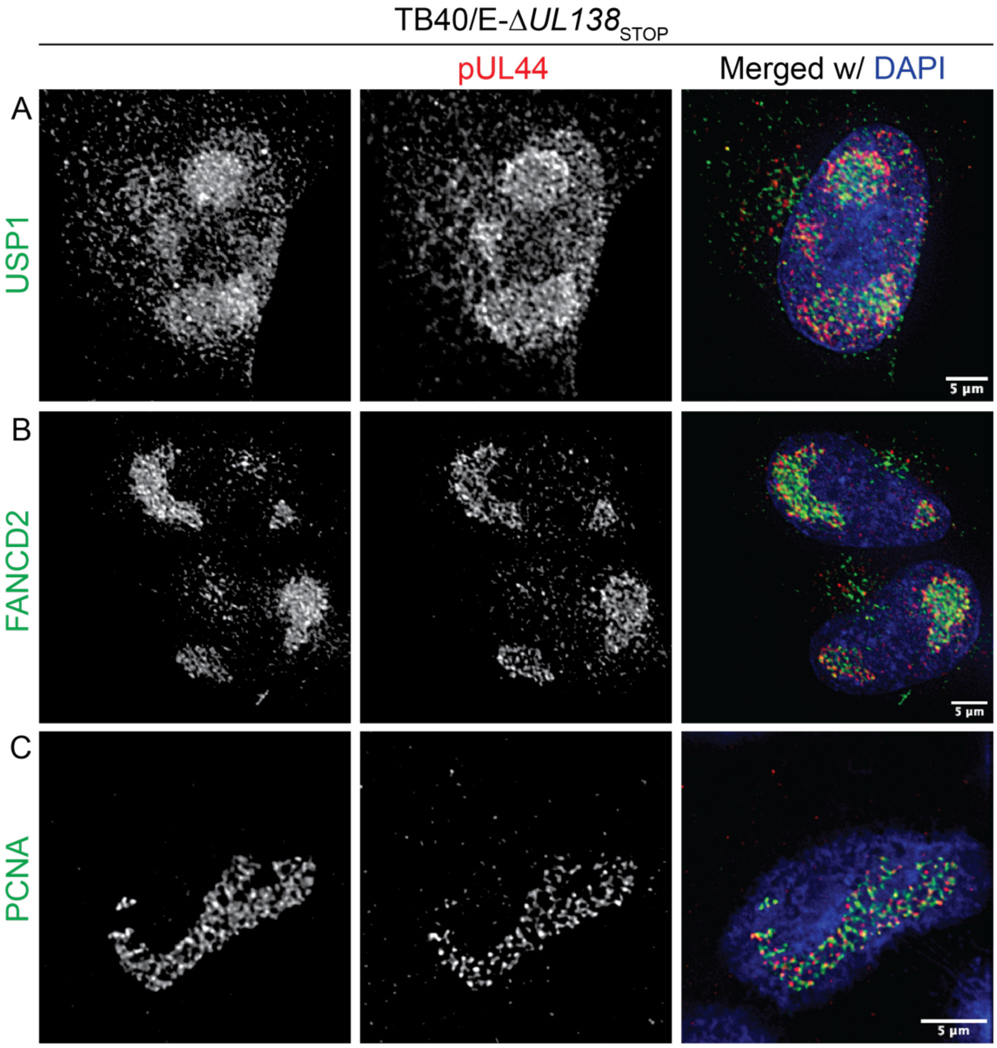

Figure S1. USP1, FANCD2, and PCNA localization to viral RCs is independent of UL138. Fibroblasts were infected with TB40/E-Δ*UL138*$_{STOP}$ at an MOI of 1. At 48 hpi cells were subject to CSK extraction and fixation and then processed for indirect immunofluorescence. (A) USP1, (B) FANCD2, and (C) PCNA were detected with pUL44 to mark sites of viral DNA synthesis using monoclonal antibodies specific to each and then secondary antibodies conjugated to Alexa Fluor® 546 (green) or 647 (red). Images were captured using a DeltaVision deconvolution microscope, and each deconvolved image corresponds to a single focal plane. Scale bar, 5 µm.

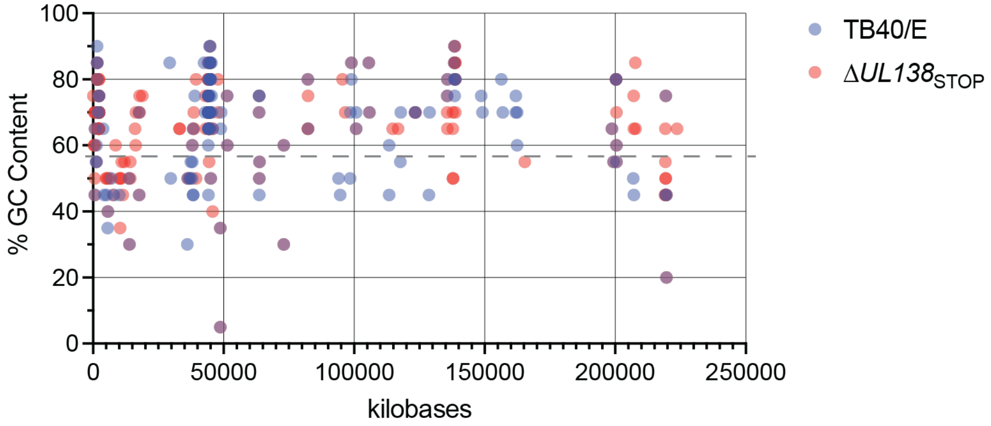

Figure S2. GC content around novel junctions arising in TB40/E-WT and -Δ*UL138*$_{\mathbf{STOP}}$ infections. GC content of 20-nucleotides on side of each novel junction is plotted for each infection (±SEM). Dotted line represents average 57% GC content of the whole HCMV genome.

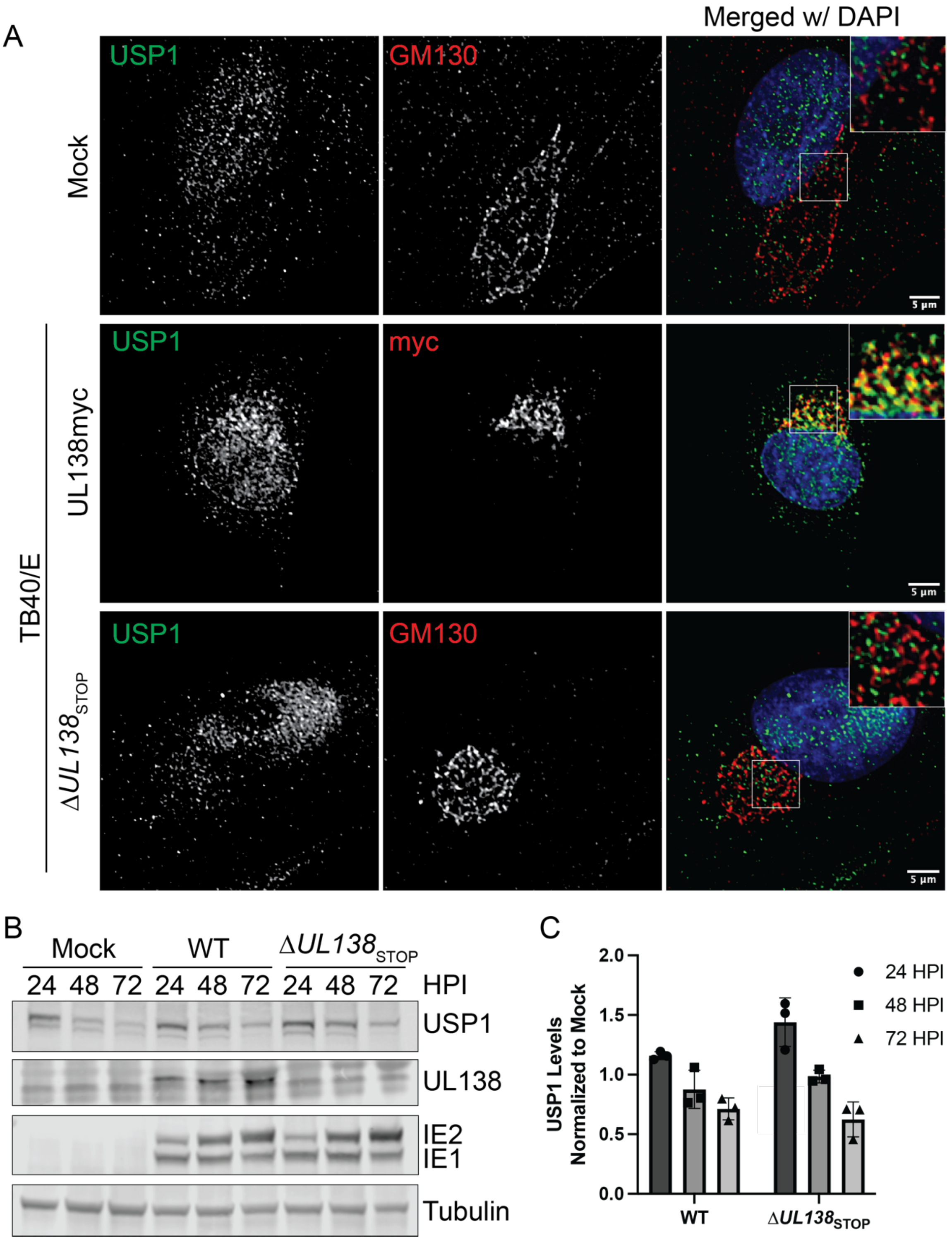

A
Merged w/ DAPI
Mock
USP1
GM130
TB40/E
UL138myc
myc
ΔUL138STOP
5 μm
B
Mock
WT
ΔUL138STOP
24 48 72 24 48 72 24 48 72
HPI
USP1
UL138
IE2
IE1
Tubulin
C
USP1 Levels Normalized to Mock
2.0
1.5
1.0
0.5
0.0
WT
ΔUL138STOP
24 HPI
48 HPI
72 HPI

Figure S3. Investigating the effect of UL138 on USP1 localization and expression during CMV infection.

(A) Fibroblasts were mock-infected or infected with TB40/E-UL138myc (C-terminal myc tag on UL138) or Δ*UL138*$_{STOP}$ at an MOI of 1. At 48 hpi, cells were fixed and processed for indirect immunofluorescence using monoclonal antibodies to USP1 and GM130 to detect the Golgi apparatus (where UL138 primarily localizes during infection) or myc to detect UL138. Secondary antibodies were conjugated to Alexa Fluor® 546 (green) or 647 (red). A magnified image is shown in the top right corner inset of the merged image. DAPI is used to mark the cell nucleus. Images were captured using a DeltaVision deconvolution microscope, and each deconvolved image corresponds to a single focal plane. Scale bar, 5 µm. (B) Fibroblasts were mock-infected or infected (MOI = 1) with TB40/E-WT or -Δ*UL138*$_{STOP}$ virus over a 72-hour time course. Immunoblotting was performed on whole cell lysates collected at the indicated time points. The indicated proteins were detected using antibodies specific to each and secondary antibodies conjugated to DyLight™ 680 (mouse) or 800 (rabbit). IE1/2 serve as markers for infection, and tubulin serves as a loading control. (C) Quantification of USP1 levels relative to tubulin and normalized to the Mock 24 hpi time point for WT and Δ*UL138*$_{STOP}$ infection.

Antibody	Species	Source	Concentration
FANCD2	Rabbit	Novus Biologicals, NB100-182	IB: 1:10,000<br>IF: 1:200
IE1/2	Mouse	Thomas Shenk, PhD<br>(Princeton University)	IB 1:100
mUb-PCNA	Rabbit	Cell Signaling Technology<br>(CST), #13439	IB 1:1000<br>IF 1:100
PCNA	Mouse	Santa Cruz, sc-56	IB 1:1000
PCNA	Rabbit	CST, #13110	IF 1:400
α-Tubulin	Mouse	Sigma-Aldrich, #T9026	IB 1:2000
pUL44	Mouse	Virusys, #CA006	IB, IF 1:12,000
UL138	Rabbit	Open Biosystems (custom)	IB, 2.4 μg/mL
USP1	Rabbit	CST, #8033	IB: 1:1000
USP1	Rabbit	LSBio, #LS-C288484	IF: 1:250
Mouse IgG (H+L) secondary, DyLight 680	Goat	Invitrogen, #35519	IB: 1:6000
Rabbit IgG (H+L) secondary, DyLight 800	Goat	Invitrogen, #SA5-10036	IB: 1:6000
Rabbit IgG (H+L) secondary, Alexa Fluor 546	Goat	Invitrogen, #A-11035	IF 1:3000
Mouse IgG (H+L) secondary, Alexa Fluor 647	Goat	Invitrogen, #A-21236	IF: 1:3000

Table S1. Antibodies used in this study.
